# ﻿New records of two roughy fish species of *Hoplostethus* and a confirmed record of *H.crassispinus* Kotlyar, 1980 (Trachichthyiformes, Trachichthyidae) from Taiwan

**DOI:** 10.3897/zookeys.1149.96233

**Published:** 2023-02-22

**Authors:** Yo Su, Hsiu-Chin Lin, Hsuan-Ching Ho

**Affiliations:** 1 Department of Marine Biotechnology and Resources, National Sun Yat-sen University, Kaohsiung, Taiwan; 2 Doctoral Degree Program in Marine Biotechnology, National Sun Yat-sen University, Kaohsiung, Taiwan; 3 National Museum of Marine Biology & Aquarium, Pingtung, Taiwan; 4 Australian Museum, Sydney, Australia; 5 Department and Graduate Institution of Aquaculture, National Kaohsiung University of Science and Technology, Kaohsiung, Taiwan

**Keywords:** Actinopterygii, biodiversity, distribution, ichthyofauna, taxonomy

## Abstract

Two rarely caught species of the roughy fish genus *Hoplostethus* have been identified for the first time in the fish collections of Taiwan. The first, *H.grandperrini* Roberts & Gomon, 2012 was previously known only from two type specimens collected in the Southern Hemisphere off the coast of New Caledonia. Its distribution is now extended to the Northern Hemisphere off the coast of Pingtung, southern Taiwan. Our specimen represents the only record of this species since its initial description. The second, *H.robustispinus* Moore & Dodd, 2010 was originally described from a single specimen collected in the Philippines and was only known from the type locality and a single record off the Paracel Islands, South China Sea. This specimen represents the third record of the species since its original description. A single specimen of *H.crassispinus* Kotlyar, 1980, whose name has long appeared in the ichthyological literature of Taiwan and adjacent areas, was also identified as the first specimen-based record for Taiwan. Detailed descriptions of these species are provided and compared with available data of respective type specimens and related species, with intraspecific variations also discussed. Also included is a dichotomous key to all known species of the subgenus Hoplostethus in Taiwan.

## ﻿Introduction

The circumglobal roughy fish genus *Hoplostethus* is the most diverse group within the family Trachichthyidae, presently comprising 30 valid species (Su et al. 2022). They are characterized by having the combination of 3–8 dorsal-fin spines, lateral-line scales distinctly enlarged, body height >40% standard length (SL), and position of anus immediately before anal-fin origin (Kotlyar 1996). The genus *Hoplostethus* has been divided into four subgenera, with the nominate subgenus Hoplostethus differing from subgenera *Aulohoplostethus*, *Leiogaster*, and *Macrohoplostethus* by having light-colored pectoral fins, simple and unbranched pyloric caeca, 25–27 total vertebrae, enlarged abdominal scutes, and no striation area on the body (Kotlyar 1986; Su et al. 2022). *Hoplostethusrobustispinus* Moore & Dodd, 2010 was described from a single specimen collected east of Calagua Islands, Philippines, and later known from another record near Paracel Islands, South China Sea (Moore and Dodd 2010; Kotlyar 2011). It is characterized by having thickened fin spines in adults, 16 or 17 pectoral-fin rays, 19 or 20 total developed gill rakers, a longer trunk (36.8–38.7% SL), 50–56 pyloric caeca, and all fins without black margin (Moore and Dodd 2010; Kotlyar 2011).

Another rarely caught species, *Hoplostethusgrandperrini* Roberts & Gomon, 2012, was described based on two specimens collected off the coast of New Caledonia (Roberts and Gomon 2012). It is distinguished from congeners by having a whitish oral cavity, 17 or 18 pectoral-fin rays, 19 or 20 total gill rakers, a short pectoral fin, the tip of which does not reach a vertical position through the anal-fin origin, and a larger maximum body size, reaching 455 mm SL (Roberts and Gomon 2012). Due to their rarity in collections, no additional information on these two species exists since their original descriptions.

*Hoplostethuscrassispinus* Kotlyar, 1980 was originally described from specimens collected from the Emperor Seamounts and was later identified based on specimens from the Kyushu-Palau ridge (Kotlyar 1980, 1986). Although this species has been recorded in Taiwan (e.g., Shen et al. 1993; Shen and Wu 2011; Koeda 2019), it has long been confused and attributed to an undescribed species (*H.* sp., in Su et al. 2022). Therefore, the existence of this species in Taiwanese waters remains unknown.

In Taiwan, five species have been recorded: *H.crassispinus*, *H.japonicus* Hilgendorf, 1879, *H.mediterraneus* Cuvier, 1829, *H.roseus* Su, Lin & Ho, 2022, and *H.* sp. (Chen 1969; Shen et al. 1993; Shen and Wu 2011; Su et al. 2022). However, three of them were only recently recognized (*H.japonicus*, *H.roseus*, and *H.* sp.; Su et al. 2022). A thorough study of the genus is needed, as new species or new records are expected (Su et al. 2022).

Recently, three specimens were found in the fish collections in Taiwan. Based on their unique characters, the three specimens are here identified as *H.robustispinus*, *H.grandperrini*, and *H.crassispinus*, respectively. Detailed descriptions of the specimens and comparisons with their respective type specimens, and available data, are herein provided. Moreover, a dichotomous identification key for all known species of the subgenus Hoplostethus (including *H.* sp., sensu Su et al. 2022) that occurs in Taiwan is also provided.

## ﻿Materials and methods

Methods for counts and measurements and description follow Su et al. (2022) except for the vertebral count, with the urostyle counted as the last vertebrae. Standard length (SL) and head length (HL) were used throughout, except where otherwise indicated. All measurements were made using digital calipers rounding to the nearest 0.1 mm, except for lengths longer than 150 mm, which were rounded to the nearest 1 mm by using a regular ruler. Paired-fin characters are presented as left/right. Counts of vertebrae and predorsal bones were determined by radiography. Specimens were deposited at the Academia Sinica, Biodiversity Research Center, Taipei, Taiwan (**ASIZP**), Fisheries Research Institute, Keelung, Taiwan (**FRIP**), and the Pisces Collection of the National Museum of Marine Biology and Aquarium, Pingtung, Taiwan (**NMMB-P**). Comparative materials including *Hoplostethusjaponicus*, *H.roseus*, and *H.* sp., are listed in Su et al. (2022).

## ﻿Results

### ﻿Family Trachichthyidae

#### ﻿ Hoplostethus

Taxon classificationAnimaliaTrachichthyiformesTrachichthyidae

Cuvier, 1829

CBA15258-643E-50D9-BFDD-5EB76CDAFDC6

Hoplostethus Cuvier in Cuvier and Valenciennes 1829: 469 (type species: Hoplostethusmediterraneus Cuvier in Cuvier and Valenciennes 1829).
Korsogaster Parr, 1933: 9 (type species: Korsogasternanus = Hoplostethusmediterraneus).

##### Remarks.

*Hoplostethus* differs from other genera of Trachichthyidae in having the following combination of characters: body depth at dorsal-fin origin >40% SL; anus situated in front of anal-fin origin; dorsal-fin spines progressively longer posteriorly and longest at last spine; lateral-line scales distinctly larger than adjacent body scales, ca 2–3 times in size; light organ absent; and vomer usually without teeth (Kotlyar 1996; Su et al. 2022).

Kotlyar (1986) divided *Hoplostethus* into four subgenera, *Aulohoplostethus*, *Hoplostethus*, *Leiogaster*, and *Macrohoplostethus*, by their numbers of dorsal-fin soft rays, pyloric caeca, and total vertebrae, coloration of pectoral fin, simple or branched pyloric caeca, and the presence of stritationed area on body. Among those subgenera, the subgenus Hoplostethus is distinguished from other subgenera in having dorsal-fin soft rays 12–14 (vs 15–19 in *Macrohoplostethus*), total vertebrae 25–26 (vs 29–30 in *Macrohoplostethus*), pyloric caeca <50, simple and unbranched (vs >60, branched in *Macrohoplostethus*), pectoral fin pale (vs black in *Leiogaster*), and body without stritationed area (vs present in *Aulohoplostethus*).

Among the 30 valid species in *Hoplostethus*, one species is recognized under *Aulohoplostethus*, 19 under *Hoplostethus*, eight under *Leiogaster*, and two under *Macrohoplostethus*, respectively.

#### ﻿ Hoplostethus grandperrini

Taxon classificationAnimaliaTrachichthyiformesTrachichthyidae

Roberts & Gomon, 2012

18D3EECE-35BF-5812-9CF3-B36B0F31F093

**Figure 1.**
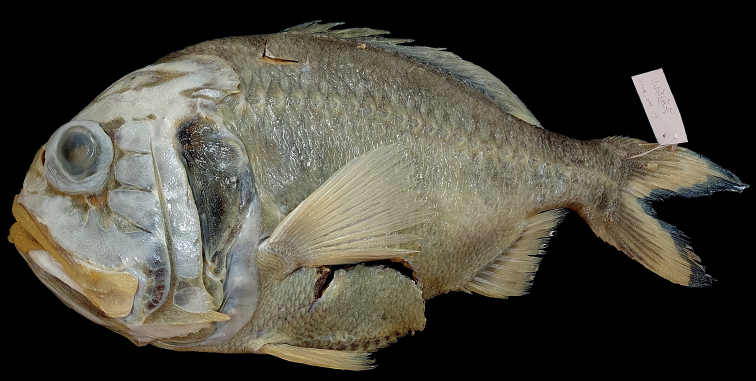
*Hoplostethusgrandperrini* Roberts & Gomon, 2012. NMMB-P36039, 395 mm SL, Pingtung, Taiwan.

**Figure 2.**
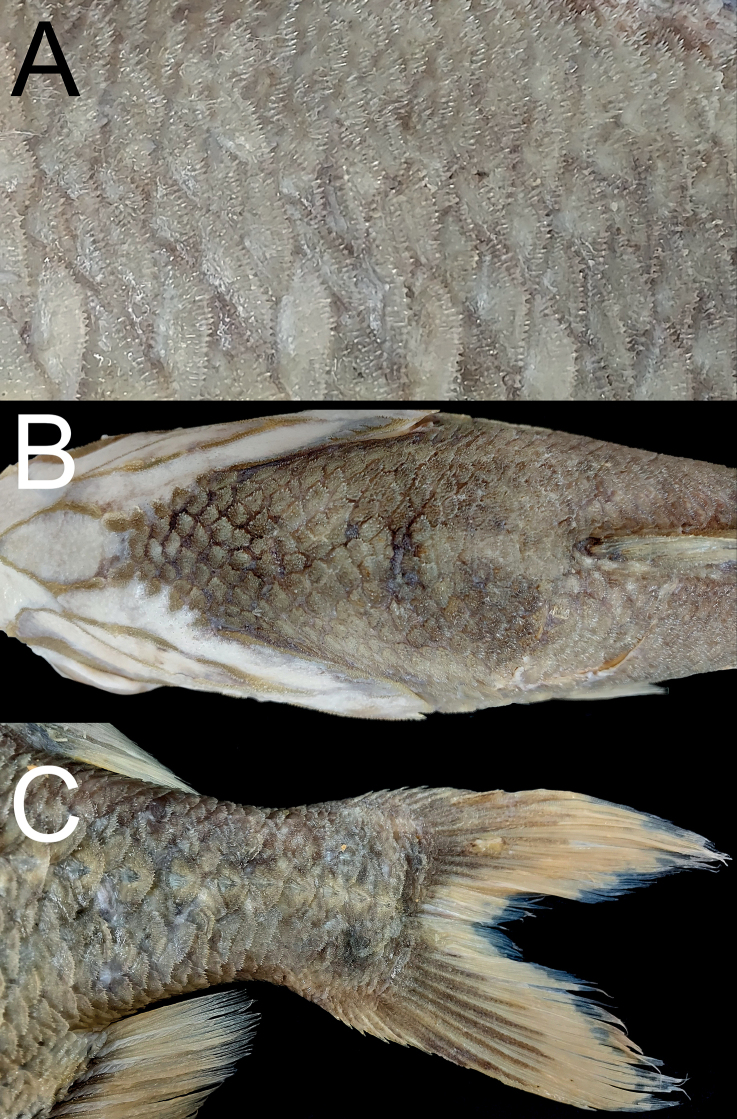
*Hoplostethusgrandperrini*, NMMB-P36039, 395 mm SL**A** scales on the dorsal side of the body featuring long spinules **B** predorsal scales **C** caudal-fin pigmentation. Figs not to scale.

**Figure 3.**
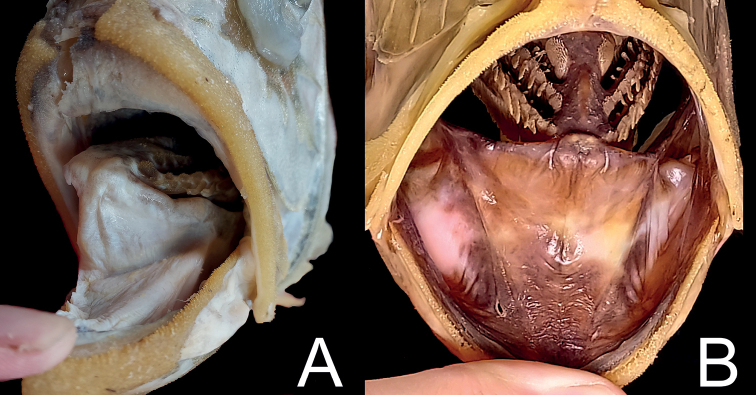
Coloration of oral cavities of *Hoplostethus* species **A***H.grandperrini*, NMMB-P36039, 395 mm SL, preserved **B***H.robustispinus*, FRIP01364, 241 mm SL, preserved.

Figs 1 , 2 , 3A ; Tables 1 , 2 English name: Grandperrin’s roughy Chinese name: 格蘭氏胸燧鯛

Hoplostethus grandperrini Roberts & Gomon, 2012: 351 (type locality: New Caledonia, Norfolk Ridge, 24°55'8.99"S, 168°20'56.99"E, depth 600–675 m).
Hoplostethus cf. gigas –Grandperrin and Lehodey 1992: 7, 26, 35.

##### Material examined.

NMMB-P36039, 395 mm SL, off the coast of Shan-hai fishing port, Pingtung, southwestern Taiwan (ca 21°59'08.75"N, 120°42'42.03"E), 19 April 2014, hook and line, purchased by C.-W. Chang.

##### Description of NMMB-P36039.

Meristic and morphometric values are provided in Tables 1, 2.

**Table 1.** Meristic data of *Hoplostethusgrandperrini* and *H.robustispinus.* Data of other specimens were obtained from Kotlyar (2011), Moore and Dodd (2010), and Roberts and Gomon (2012). Paired-fin characters are presented as left/right.

	*Hoplostethusgrandperrini*		*Hoplostethusrobustispinus*		
	This study	Roberts and Gomon (2012)	This study	Moore and Dodd (2010)	Kotlyar (2011)
	NMMB-P36039	All types (*n* = 2)	FRIP 01364	Holotype	ZMMU-P22657
Dorsal-fin elements	VI, 13	VI, 13	VI, 13	VI, 14	VI, 13
Pectoral-fin elements	17/17	17–18	17/17	16–17	16/16
Pelvic-fin elements	I, 6/I, 6	I, 6	I, 6/I, 6	I, 6	N/A
Anal-fin elements	III, 9	III, 9	III, 9	III, 9	III, 9
Gill rakers	6+1+13=20	6+1+12–13=19–20	6+1+12=19	6+1+13=20	6+1+12=19
Pseudobranchial filaments	30	15 (n=1)	26	25–26	27–28
Lateral-line scales	28	29	28	28	28
Scale rows above lateral line	13	11–12	10	N/A	N/A
Scale rows below lateral line	23	26–35	27	N/A	N/A
Abdominal scutes	17	13–14	15	13	14
Predorsal scales	24	21–24	23	23	22
Pyloric caeca	44	N/A	N/A	56	50
Vertebrae	11+16	11+15	11+16	11+16	11+16

**Table 2.** Morphometric data of *Hoplostethusgrandperrini* and *H.robustispinus*. Data of other specimens were obtained from Kotlyar (2011), Moore and Dodd (2010), and Roberts and Gomon (2012). Abbreviations: A, anal-fin; C, caudal-fin; D, dorsal-fin; GR, gill raker; HF, forehead height; HL, head length; P, pectoral-fin; V, pelvic-fin.

	*Hoplostethusgrandperrini*			*Hoplostethusrobustispinus*			
	This study		Roberts and Gomon (2012)	This study		Moore and Dodd (2010)	Kotlyar (2011)
	NMMB-P36039		All types (*n* = 2)	FRIP 01364		Holotype	ZMMU-P22657
SL (mm)	395		131–455	241		340	163
	%SL	%HL	%SL	%SL	%HL	%SL	%SL
HL	37.6		41.4–42.9	38.0		35.7	38.7
Head depth	39.5	105.2	N/A	37.8	99.3	40.1	43.5
Predorsal length	46.5	123.7	38.8–49.1	45.1	118.6	44.1	46.3
Prepectoral length	39.8	105.9	40.4–40.8	36.6	96.2	37.1	40.8
Prepelvic length	42.0	111.8	42.6–43.8	39.5	103.8	42.6	46.3
Preanal length	67.6	180.0	73.2–76.7	72.6	190.8	69.6	76.1
Snout length	8.2	21.8	9.5–10.5	7.8	20.5	10.0	9.3
Eye diameter	10.1	26.8	9.6–12.5	11.0	28.8	9.4	11.7
Interorbital length	13.6	36.3	13.4–14.1	12.0	31.6	11.2	11.7
Upper-jaw length	25.6	68.1	26.3–30.4	25.9	68.2	24.9	26.1
Lower-jaw length	26.8	71.3	28.6–31.1	26.2	68.8	24.2	26.1
HF1	3.2	8.4	N/A	4.2	11.0	N/A	3.7
HF2	6.7	17.8	41.0–44.2	6.1	16.1	N/A	N/A
Postorbital length	18.5	49.3	22.0–22.4	19.6	51.4	18.8	18.4
P length	24.8	66.1	25.2–34.0	33.2	87.4	27.8	28.2
D–P length	32.9	87.5	N/A	36.0	94.5	N/A	N/A
D–V length	48.8	130.0	N/A	46.2	121.4	N/A	N/A
Body height	49.4	131.4	53.2–55.6	47.7	125.3	46.4	50.9
V length	18.5	49.4	N/A	21.3	56.1	19.5	23.0
V spine length	13.9	36.9	N/A	16.4	43.1	N/A	N/A
P–V length	14.3	38.1	15.8–18.3	10.7	28.0	N/A	10.4
D–A length	49.5	131.7	N/A	50.5	132.6	N/A	N/A
V–A length	32.1	85.6	35.5–42.4	39.3	103.4	38.7	36.8
D length	38.2	101.7	36.0–38.3	36.8	96.7	37.9	40.5
D height	18.0	48.0	N/A	broken		N/A	N/A
1^st^ D spine length	4.9	12.9	2.9–4.7	4.3	11.3	N/A	N/A
2^nd^ D spine length	7.2	19.1	4.1–8.9	7.0	18.5	N/A	N/A
Last D spine length	12.4	32.9	12.1–17.6	16.9	44.3	N/A	N/A
A length	15.8	42.1	15.8–18.3	17.8	46.7	16.1	19.0
A height	15.0	39.8	N/A	12.7	33.3	N/A	N/A
3^rd^ A spine length	9.7	25.7	7.7–13.3	12.4	32.6	N/A	N/A
Postanal length	23.1	61.5	N/A	22.5	59.1	N/A	20.9
Postdorsal length	27.3	72.6	N/A	22.7	59.8	N/A	23.9
Caudal-peduncle height	11.5	30.6	12.5–13.0	11.1	29.2	11.6	9.6
C length	25.4	67.5	N/A	broken		N/A	N/A
Longest GR	7.1	18.9	N/A	6.7	17.6	7.0	7.4
Gill filament at angle	2.4	6.3	N/A	1.9	4.9	N/A	N/A
Longest pseudobranchial filament	4.4	11.6	N/A	3.8	10.0	N/A	4.3

Dorsal-fin rays VI, 13; pectoral-fin rays 17/17; pelvic-fin rays I, 6/I, 6; anal-fin rays III, 9; principal caudal-fin rays 10+9=19, uppermost and lowermost rays unbranched; procurrent caudal-fin rays 7 dorsally and 7 ventrally; gill rakers on outer surface of first-gill arch 6+1+13=20; lateral-line scales 28; scale rows between dorsal-fin origin and lateral line 13, scale rows between anal-fin origin and lateral line 23; predorsal scales 24; abdominal scutes 17; vertebrae 11+16=27; pyloric caeca 44; pseudobranchial filaments 30; branchiostegal rays 8; supraneural and pterygiophore insertion formula: 0/0/2+1/1/1/1 (spinous dorsal fin only).

Body oblong, distinctly longer than deep, depth at dorsal-fin origin 2.0 in SL. Head large, its length 2.7 in SL, its height subequal to its length, 1.0 in HL; upper profile in front of dorsal fin slightly curved to back of head, with somewhat concave forehead, and abrupt downturn above maxilla; forehead broad, HF1 11.9 and HF2 5.6 in HL; eyes of moderate size, 3.7 in HL; snout length 4.6 in HL; space between eyes convex and broad, interorbital width 2.8 in HL; crests on head bones well developed and covered with rather long spinules.

Mouth large, posterior end of maxilla extending beyond vertical through posterior margin of eye. Nostrils right before anterior margin of eye, on horizontal about through center of eye; posterior nostril distinctly larger than anterior nostril; eyes rather ventrally placed, upper margin of eye on horizontal through lateral-line origin.

Most of lateral and medial surfaces of premaxilla and dentary covered with villiform teeth, those on medial surface rather conical; no teeth at symphyseal notch of premaxilla and knob at symphysis of dentaries. Narrow band of villiform teeth on palatine; vomer toothless. Gill rakers on first and second arch rod-shaped, laterally compressed, with small conical teeth on tips and inner surfaces; those in outer row of first arch longest; those on inner row of first arch and both inner and outer rows of second and third arches short; small tooth patches, forming bumps on midline of outer three arches; large tooth patches present on fourth ceratobranchial arch. Large, oval patch of villiform teeth on fifth ceratobranchial. Large, slightly oval tooth patch on second pharyngobranchial. Large teardrop-like tooth patch on third pharyngeal arch.

Preopercular spine short, its tip not reaching pelvic-fin base. Longest gill raker 0.7 in eye diameter; gill filaments at angle of first gill arch very short, ca 4.3 in eye diameter, and ca 1.8 in length of longest pseudobranchial filaments.

Body scales firmly attached, cycloid scales present on pectoral-fin region, elsewhere covered with ctenoid scales armored with rather long spinules (Fig. 2A); isthmus and gular region naked; lateral-line scales enlarged, ca 2–3 times size of body scales; center of each lateral-line scale without distinct spine; enlarged scales (scutes) covering abdomen region, their bases covered with body scales, all scutes with single tip; predorsal scales not enlarged and not forming distinct ridge (Fig. 2B).

Dorsal-fin spines progressively longer posteriorly, greatest increase in length from first to fourth spine; first ray unbranched, others branched; outer margin of dorsal-fin rays nearly straight. Pectoral fin truncated, slightly rounded; short, not reaching vertical through anal-fin origin. Pelvic fin short, reaching 11^th^ abdominal scute. Pyloric caeca pale, unbranched.

##### Coloration.

Fresh coloration unknown, presumably a reddish body color as shown by Roberts and Gomon (2012: fig. 6). Preserved specimen uniformly pale, slightly yellowish (Fig. 1), membranes on head region lighter than body color. Anal- and caudal-fin rays with black pigmentation near base (Fig. 2C). Oral cavity, including underside of tongue white, with very scarce black pigmentation (Fig. 3A). Inner side of opercle black. Peritoneum and stomach black.

##### Distribution.

Previously, only type series collected between 600 and 675 m deep off the coast of New Caledonia were known. Our specimen represents the second record and a range extension to the Northern Hemisphere, suggesting a wide distribution in the western Pacific Ocean.

##### Remarks.

The present specimen was identified as *H.grandperrini* by having a pale oral cavity, a short pectoral fin with its tip not reaching a vertical through the anal-fin origin, predorsal scales not enlarged and forming a distinct ridge, head bones covered with long spinules, ctenoid scales on body with rather long spinules, and a larger size, exceeding 300 mm SL (Roberts and Gomon 2012).

*Hoplostethusgrandperrini* can be distinguished from other species of *Hoplostethus* co-occurring in Taiwan by having the following characters: a pale oral cavity, including the underside of the tongue (vs uniformly black oral cavity in both *H.japonicus* and *H.roseus*), a short pectoral fin with its tip not reaching a vertical through the anal-fin origin (vs a long pectoral fin exceeding beyond a vertical through the anal-fin origin in *H.japonicus* and *H.* sp.; sensu Su et al. 2022).

In comparison to the data provided by Roberts and Gomon (2012), our specimen has a higher number of abdominal scutes (17, vs 13–14) and pseudobranchial filaments (30, vs 15), a slightly lower number of lateral-line scales (28, vs 29) and fewer scale rows below the lateral line (23, vs 26–35), and some slightly different morphometric characters (e.g., smaller head length, prepectoral length, and prepelvic length; Tables 1, 2). These values are rather distinct, and additional research may reveal whether the Taiwanese population represents a different species. It is also notable that the forehead length (HF2) of our species is 6.7%, distinctly different from 41.0–44.2% SL provided by Roberts and Gomon (2012). Based on our previous data (e.g., 3.7–7.9% SL in specimens examined by Su et al. 2022), it is likely that they were referring to “head height” rather than “forehead height”.

#### ﻿ Hoplostethus robustispinus

Taxon classificationAnimaliaTrachichthyiformesTrachichthyidae

Moore & Dodd, 2010

EE33A2E8-EDAE-5471-9594-BF527D9069F1

**Figure 4.**
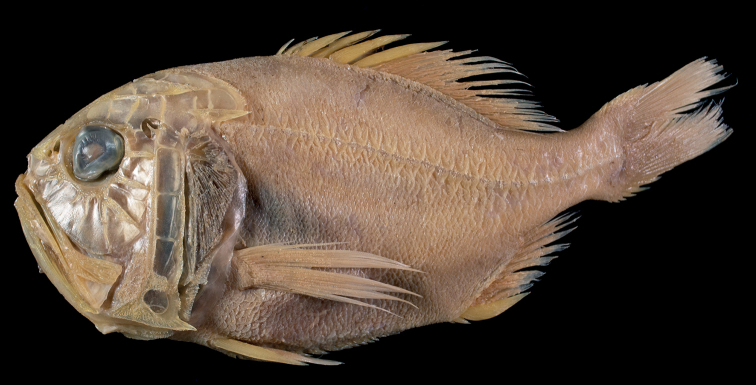
*Hoplostethusrobustispinus* Moore & Dodd, 2010, FRIP 01364, 241 mm SL, South China Sea, Taiwan.

**Figure 5.**
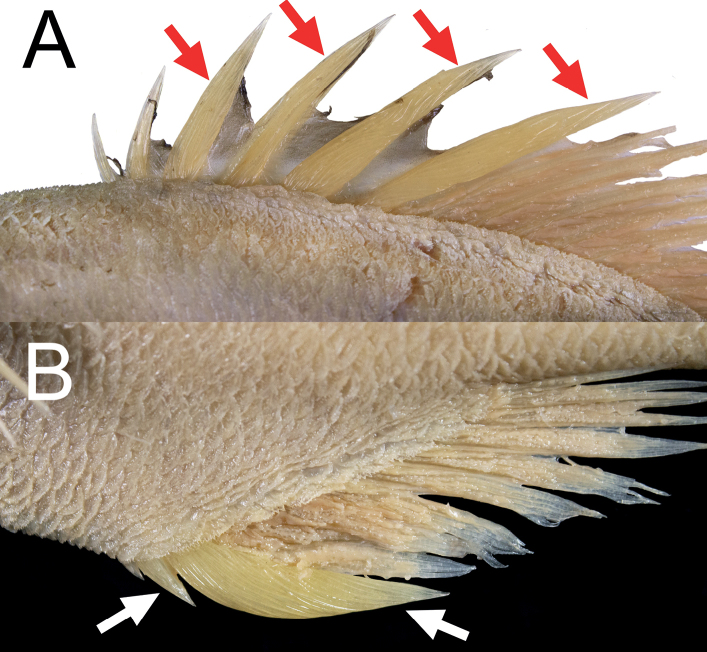
*Hoplostethusrobustispinus*, FRIP 01364, 241 mm SL, showing thickened dorsal- (**A**) and anal- (**B**) fin spines (arrowed), and coloration of membranes between dorsal-fin spines (**A**).

Figs 3B–3 , 4 , 5 ; Tables 1 , 2 English name: Thickspine roughy Chinese name: 粗棘胸燧鯛

Hoplostethus robustispinus Moore & Dodd, 2010: 139 (type locality: east of Calagua Islands, Philippines, 14°18'00"N–14°47'00"N, 123°21'00"E–123°25'00"E, depth 648–660 m)–Kotlyar 2011: 484 (14°34'00"N, 112°06'00"E, South China Sea, depth 300 m).

##### Material examined.

FRIP 01364, 241 mm SL, South China Sea, 30 April 1996, collected by D.-A. Lee.

##### Description of FRIP 01364.

Meristic and morphometric data are provided in Tables 1, 2.

Dorsal-fin rays VI, 13; pectoral-fin rays 17/17; pelvic-fin rays I, 6/I, 6; anal-fin rays III, 9; principal caudal-fin rays 10+9=19, uppermost and lowermost rays unbranched; procurrent caudal-fin rays 7 dorsally and 7 ventrally; gill rakers on outer surface of first gill arch 6+1+12=19; lateral-line scales 28; scale rows between dorsal-fin origin and lateral line 10, scale rows between and anal-fin origin and lateral line 27; predorsal scales 23; abdominal scutes 15; vertebrae 11+16=27; pseudobranchial filaments 26; branchiostegal rays 8; supraneural and pterygiophore insertion formula: 0/0/2+1/1/1/1 (spinous dorsal fin only).

Body oblong, distinctly longer than deep, depth at dorsal-fin origin 2.1 in SL. Trunk large, length from pelvic-fin origin to anal-fin origin 2.5 in SL. Head large, its length 2.6 in SL, its height subequal to its length, 1.0 in HL; upper profile in front of dorsal fin rather flat, slightly curved to back of head, with somewhat rounded forehead, and abrupt downturn above maxilla; forehead broad, HF1 9.1 and HF2 6.2 in HL; eyes of moderate size, 3.5 in HL; snout length 4.9 in HL; space between eyes convex and broad, interorbital width 3.2 in HL; crests on head bones well developed and covered with small spinules.

Mouth large, posterior end of maxilla reaching vertical through posterior margin of eye. Nostrils right before anterior margin of eye, slightly above horizontal through center of eye; posterior nostril distinctly larger than anterior nostril; eyes rather dorsally placed, upper margin of eye on horizontal through lateral-line origin.

Most of lateral and medial surfaces of premaxilla and dentary covered with villiform teeth, those on medial surface rather conical; no teeth at symphyseal notch of premaxilla and knob at symphysis of dentaries. Narrow band of villiform teeth on palatine; vomer toothless. Gill rakers on first and second arch rod-shaped, laterally compressed; those in outer row of first arch longest; those on inner row of first arch and both inner and outer rows of second to fourth arches short.

Preopercular spine short, its tip not reaching pelvic-fin base. Longest gill raker 1.6 in eye diameter; gill filaments at angle of first gill arch very short, ca 5.9 in eye diameter, and ca 2.0 in length of longest pseudobranchial filaments.

Body scales firmly attached, cycloid scales present on pectoral-fin region, elsewhere covered with ctenoid scales; isthmus and gular region naked; lateral-line scales enlarged, ca 2–3 times size of body scales; center of each lateral-line scale without distinct spine; enlarged scales (scutes) covering abdomen region, their bases covered with body scales, all scutes with single tip; predorsal scales slightly enlarged and forming ridge.

Dorsal-fin spines progressively longer posteriorly, greatest increase in length from first to third spine; third to sixth spine extremely thickened, greatest width 5.6–8.9 in its length (Fig. 5A); first ray unbranched, others branched; outer margin of dorsal-fin rays nearly straight. Pectoral fin truncated, slightly rounded; short, not reaching vertical through anal-fin origin. Pelvic fin short, reaching eighth abdominal scute; its spine slightly thickened, greatest width 8.8 times in its length. Second and third anal-fin spines extremely thickened, greatest width 3.5–4.8 times in its length (Fig. 5B).

##### Coloration.

Fresh condition of our specimen unknown, presumably a uniformly bright-red coloration as shown in Moore and Dodd (2010: fig. 3). Preserved specimen (Fig. 4) uniformly yellowish-brown, all fin spines paler than body color; fin-ray color similar to body color. Distal half of membrane between dorsal-fin spines black (Fig. 5A). Oral cavity, including underside of tongue mostly black, with some portions slightly whitish (Fig. 3B); inner side of opercle, and peritoneum black.

##### Distribution.

Originally described from the Philippine Sea (Moore and Dodd 2010), and a later record reported from south to Paracel Islands in the South China Sea (14°34'00"N, 112°06'00"E) (Kotlyar 2011). Although the precise location of our specimen is unknown, it is presumed to be in the northern portion of the South China Sea, most likely near the Dong-sha Islands (Pratas Islands). Our specimen represents the third published record of the species and the first in Taiwan.

##### Remarks.

Our specimen was identified as *H.robustispinus* by having thickened fin spines, a short pectoral fin that does not reach the vertical through the anal-fin origin, and a black oral cavity (Moore and Dodd 2010). *Hoplostethusrobustispinus* can be separated from other co-occurring species in Taiwan based on its thickened fin spines, a distinct character for this species.

Furthermore, our specimen has minor differences in meristic characters and body proportions compared to all known specimens (Moore and Dodd 2010; Kotlyar 2011) (Tables 1, 2), all of which can be attributed to intraspecific variation. Notably, it was stated that the holotype of *H.robustispinus* lacks black pigmentation on the membranes of the spinous dorsal fin, and Kotlyar (2011) stated that his specimen was reported as light-colored in his previous publication (Kotlyar 1986). However, our specimen possesses this character (Fig. 5A). This character must be confirmed when additional specimens are available.

#### ﻿ Hoplostethus crassispinus

Taxon classificationAnimaliaTrachichthyiformesTrachichthyidae

Kotlyar, 1980

29C5D38B-6FFC-5E74-9D65-A52D6B123B54

**Figure 6.**
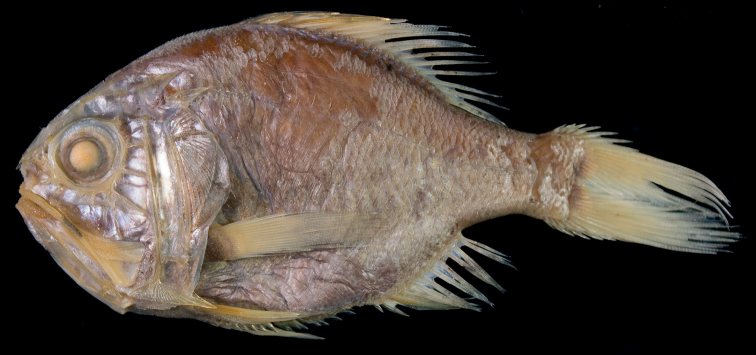
*Hoplostethuscrassispinus* Kotlyar, 1980, ASIZP0065017, 86.3 mm SL, Yilan, Taiwan.

**Figure 7.**
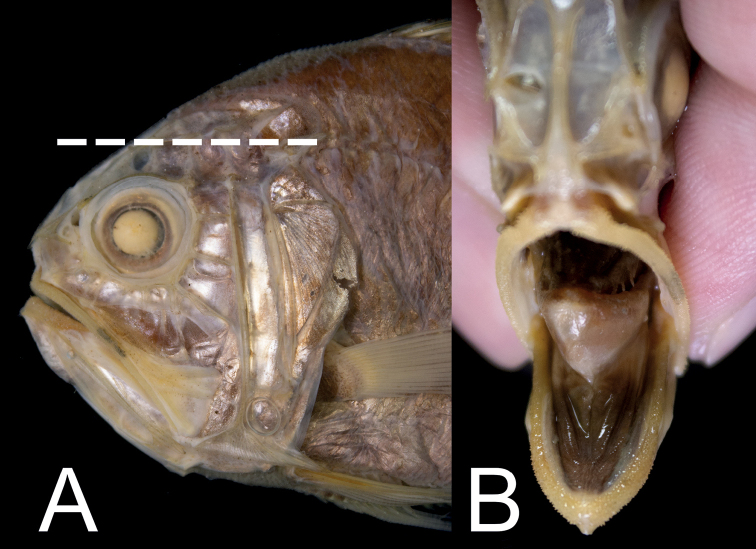
*Hoplostethuscrassispinus*, ASIZP0065017, 86.3 mm SL**A** relative position of the eye and the horizontal level of lateral-line origin (white dashed line) **B** coloration of the oral cavity. Images not to scale.

Figs 6 , 7 ; Tables 3 , 4 Chinese name: 重胸燧鯛

Hoplostethus crassispinus Kotlyar, 1980: 1055 (type locality: Emperor Seamount Chain, Northwest Pacific, 31°05'00"N–32°01'00"N, 173°10'00"E–175°55'00"E, depth 280–360 m)–Kotlyar 1986: 127 (new record from Kyushu-Palau Ridge, 25°08'00"N, 135°41'10"E, depth 560–600 m); Kotlyar 1996: 152; Moore and Dodd 2010: 138; Kotlyar 2011: 152; Roberts and Gomon 2012: 341; Su et al. 2022: 10.
Hoplostethus sp.–Koeda et al. 2021: 17, fig. 5H (Ritto Seamount, western Mariana Ridge, western Pacific, 21°37'00"N–21°57'00"N, 141°53'00"E–142°13'00"E, depth 538 m).

##### Material examined.

ASIZP 0065017, 86.3 mm SL, off the coast of Nanfang-ao fishing port, Yilan, northeastern Taiwan (ca 24°34'53.16"N, 121°52'12.21"E), 27 June 2004, bottom trawl, collected by H.-C. Ho.

##### Description of ASIZP 0065017.

Meristic and morphometric data are provided in Tables 3, 4.

**Table 3.** Meristic data of *Hoplostehuscrassispinus*. Data of type and other specimens were obtained from Kotlyar (1980) and Kotlyar (1986). Paired-fin characters are presented as left/right.

	*Hoplostethuscrassispinus*		
	This study	Kotlyar (1980)	Kotlyar (1986)
	ASIZP 0065017	Types and non-types	All specimens (*n* = 16)
Dorsal-fin elements	VI, 13	VI–VII, 12–13	VI–VII, 12–13
Pectoral-fin elements	17/15	16–17	15–17
Pelvic-fin elements	I, 6/I, 6	I, 6	I, 6
Anal-fin elements	III, 9	III, 9	III–IV, 8–9
Gill rakers	5+1+12=18	6+1+11–13=18–20	6+1+11–13=18–20
Pseudobranchial filaments	27	N/A	23–28
Lateral-line scales	28	27–29	28–30
Abdominal scutes	15	11–15	10–15
Predorsal scales	23	N/A	21–23
Pyloric caeca	N/A	40–50	40–50
Vertebrae	11+16=27	10+16=26	10+16=26

**Table 4.** Morphometric data of *Hoplostethuscrassispinus*. Data of type and other specimens were obtained from Kotlyar (1980) and Kotlyar (1986). Abbreviations: A, anal-fin; C, caudal-fin; D, dorsal-fin; GR, gill raker; HF, forehead height; HL, head length; HT, holotype; NT, non-type; P, pectoral-fin; V, pelvic-fin.

	This study		Kotlyar (1980)	Kotlyar (1986)
	ASIZP 0065017		HT (PT; NT) (*n* = 5)	All specimens (*n* = 16)
SL (mm)	89.3		192 (136–161)	135–254
	%SL	%HL	%SL	%SL
HL	37.5		37.5 (36.0–37.1)	34.3–37.8
Head depth	40.6	108.1	41.6 (37.8–40.0)	37.8–44.0
Predorsal length	47.4	126.2	48.9 (46.4–47.5)	45.3–50.4
Prepectoral length	35.4	94.3	36.4 (36.8–39.2)	36.4–40.4
Prepelvic length	36.1	96.2	41.1 (40.4–45.0)	38.3–45.0
Preanal length	64.5	171.7	66.6 (66.9–73.3)	66.6–74.6
Snout length	7.2	19.1	9.4 (8.8–10.7)	7.9–10.7
Eye diameter	12.9	34.3	10.9 (11.0–11.4)	9.6–13.1
Interorbital length	11.5	30.7	11.4 (9.9–11.0)	9.9–12.0
Upper-jaw length	26.5	70.5	26.5 (24.4–26.0)	24.2–27.4
Lower-jaw length	26.7	71.1	27.0 (24.4–26.4)	24.2–27.8
HF1	4.0	10.6	3.1 (2.8–3.7)	2.5–4.5
HF2	6.2	16.6	N/A	N/A
Postorbital length	18.8	50.1	17.2 (14.7–16.3)	14.6–17.4
P length	30.6	81.4	30.2 (26.4–30.1)	25.6–30.9
D–P length	36.5	97.3	N/A	N/A
D–V length	50.5	134.6	N/A	N/A
Body height	52.4	139.5	51.5 (47.8–51.4)	47.8–53.7
V length	25.1	66.9	21.9 (18.6–22.5)	N/A
V spine length	18.7	49.8	N/A	N/A
P–V length	11.0	29.3	10.9 (8.8–11.2)	8.8–12.6
D–A length	54.4	144.9	N/A	N/A
V–A length	32.2	85.8	29.1 (27.1–37.3)	27.1–37.9
D length	40.7	108.4	40.0 (37.8–40.0)	37.0–42.2
D height	21.2	56.6	N/A	N/A
1^st^ D spine length	7.9	20.9	N/A	N/A
2^nd^ D spine length	broken		N/A	N/A
Last D spine length	20.2	53.8	N/A	N/A
A length	19.6	52.3	20.8 (18.6–20.6)	17.7–20.8
A height	16.1	42.8	N/A	N/A
3^rd^ A spine length	14.2	37.7	N/A	N/A
Postanal length	24.6	65.6	24.4 (21.4–24.2)	N/A
Postdorsal length	27.9	74.3	25.4 (24.3–25.7)	N/A
Caudal-peduncle height	13.4	35.8	14.0 (13.6–14.0)	N/A
C length	35.5	94.5	N/A	N/A
Longest GR	9.0	23.9	6.8 (6.2–6.6)	5.1–9.9
Gill filament at angle	2.1	5.5	N/A	N/A
Longest pseudobranchial filament	3.9	10.5	N/A	3.5–4.2

Dorsal-fin rays VI, 13; pectoral-fin rays 17/15; pelvic-fin rays I, 6/I, 6; anal-fin rays III, 9; principal caudal-fin rays 10+9, uppermost and lowermost rays unbranched; procurrent caudal-fin rays 7 dorsally and 7 ventrally; gill rakers on outer surface of first gill arch 5+1+12=18; lateral-line scales 28; predorsal scales 23; abdominal scutes 15; vertebrae 11+16=27; pseudobranchial filaments 24; branchiostegal rays 8; supraneural and pterygiophore insertion formula: 0/0/2+1/1/1/1 (spinous dorsal fin only).

Body oblong, distinctly longer than deep, depth at dorsal-fin origin 1.9 in SL. Head large, its length 2.7% in SL, its height slightly smaller than its length, 0.9% in HL; upper profile in front of dorsal fin rounded, slightly curved to back of head, with somewhat rounded forehead, and abrupt downturn above maxilla; forehead broad, HF1 9.4 and HF2 6.0 in HL; eyes rather large, 2.9 in HL; snout length 5.2 in HL; space between eyes convex and broad, interorbital width 3.3 in HL; crests on head bones well developed and covered with very tiny spinules.

Mouth large, posterior end of maxilla slightly reaching beyond vertical through posterior margin of eye. Nostrils right before anterior margin of eye, slightly higher than horizontal through center of eye; posterior nostril distinctly larger than anterior nostril; eyes rather ventrally placed, upper margin of eye distinctly lower than horizontal through lateral-line origin (Fig. 7A).

Most of lateral and medial surfaces of premaxilla and dentary covered with villiform teeth; no teeth at symphyseal notch of premaxilla and knob at symphysis of dentaries. Narrow band of villiform teeth on palatine; vomer toothless. Gill rakers on first and second arch rod-shaped, laterally compressed; those in outer row of first arch longest; those on inner row of first arch and both inner and outer rows of second to fourth arches short.

Preopercular spine rather long, its tip reaching pelvic-fin base. Longest gill raker 1.4 in eye diameter; gill filaments at angle of first gill arch very short, ca 6.2 in eye diameter, and ca 1.9 in length of longest pseudobranchial filaments.

Body scales firmly attached, cycloid scales present on pectoral-fin region, elsewhere covered with ctenoid scales; isthmus and gular region naked; lateral-line scales enlarged, ca 2–3 times size of body scales; center of each lateral-line scale without distinct spine; enlarged scales (scutes) covering abdomen region, their bases covered with body scales, all scutes with single tip; predorsal scales enlarged and forming ridge.

Dorsal-fin spines progressively longer posteriorly, greatest increase in length from first to third spine; first ray unbranched, others branched; outer margin of dorsal-fin rays nearly straight. Pectoral fin truncated, slightly rounded; rather long, reaching to third anal-fin spine. Pelvic fin rather long, reaching 14^th^ abdominal scute.

##### Coloration.

Preserved specimen yellowish-brown (Fig. 6); all fin spines paler than body color. Membranes between dorsal-fin spines with black pigmentations. Oral cavity, including underside of tongue mostly black, with some portions slightly whitish (Fig. 7B). Inner side of opercle and peritoneum black. A recent study documented a reddish body color with a silvery abdomen (Koeda et al. 2021: 10).

##### Distribution.

Originally described from the Emperor Seamount Chain (Kotlyar 1980) and subsequently recorded from the Kyushu-Palau Ridge (Kotlyar 1986). Although Kotlyar (2011) stated that this species may be restricted to these areas, our specimen confirmed the species’ westward extension.

##### Remarks.

The present specimen is identified as *H.crassispinus* by having a lower eye position, with the upper margin of the eye distinctly below the horizontal through lateral-line origin, a moderately long pectoral fin, with its end slightly exceeding the vertical through anal-fin origin, a blackish oral cavity, including the underside of the tongue, 15 or 17 pectoral-fin rays, and 18 total gill rakers. It can be distinguished from other Taiwanese species by its ventrally positioned eye, the upper margin of which is clearly below the horizontal through lateral-line origin (vs a more dorsally placed eye, the upper margin of the eye at the same horizontal through lateral-line origin in all species in Taiwan).

Although this species has long been thought to be part of the ichthyofauna of Taiwan (e.g., Shen et al. 1993; Shen and Wu 2011; Koeda 2019), the figures of these studies treated as “*H.crassispinus*” appear to be an undescribed species (*H.* sp., in Su et al. 2022). *Hoplostethuscrassispinus* differs from *H.* sp. in having total gill rakers 18–20 (vs 20–22 in *H.* sp.); predorsal scales 21–23 (vs 15–19); pyloric caeca 40–50 (vs 36); oral cavity blackish in adults (vs oral cavity whitish in adults); upper margin of the eye clearly below the horizontal through lateral-line origin (vs at the same horizontal level); gular region naked (vs gular region covered with ctenoid scales). As mentioned previously, the taxonomic study by Dr M. Gomon is ongoing, and we will await the publication of his findings (Su et al. 2022).

Compared to the morphological data provided by Kotlyar (1980, 1986), our specimen has very slight variations. Due to the fact that all other specimens are significantly larger than ours (135–254 vs 86.3 mm SL), these differences can be attributed to intraspecific variation. We discovered that an additional 136 mm SL specimen was used in Kotlyar’s (1980) description, but did not appear as a registered specimen anywhere in the article; therefore, this specimen is considered non-type material.

### ﻿Key to species of the subgenus Hoplostethus in Taiwan

**Table:** 

1	Tip of caudal fin blackish	***H.japonicus***
–	Tip of caudal fin without or with very little black pigmentation	**2**
2	Upper margin of eye below a horizontal through the lateral-line origin	***H.crassispinus***
–	Upper margin of eye on a horizontal through the lateral-line origin	**3**
3	Predorsal scales with the same size as adjacent body scales	***H.grandperrini***
–	Predorsal scales enlarged, forming a distinct ridge	**4**
4	Gular region covered with scales; oral cavity pale in adults	***H.* sp. (sensu Su et al. 2022)**
–	Gular region naked; oral cavity blackish in adults	**5**
5	Snout length 7.8–10.0% SL; caudal-fin base without brownish margin	***H.robustispinus***
–	Snout length 6.7–7.4% SL; caudal-fin base with brownish margin	***H.roseus***

## Supplementary Material

XML Treatment for Hoplostethus

XML Treatment for Hoplostethus grandperrini

XML Treatment for Hoplostethus robustispinus

XML Treatment for Hoplostethus crassispinus
